# ST36 acupoint injection with anisodamine for postoperative nausea and vomiting in female patients after bariatric surgery: a prospective, randomized controlled trial

**DOI:** 10.1007/s00464-023-10037-6

**Published:** 2023-04-24

**Authors:** Qi Xue, Qijing Xing, Ling Dong, Min Guo, Xiaoyan Zhang, Xinchun Wei, Benli Jia, Yong Wang, Hong Chen, Xianwen Hu, Hong Liu, Ye Zhang, Gordon Tin Chun Wong, Chunxia Huang

**Affiliations:** 1 Department of Anesthesiology and Perioperative Medicine, The Second Affiliated Hospital of Anhui Medical University, Hefei City, Anhui Province China; 2 Key Laboratory of Anesthesiology and Perioperative Medicine of Anhui Higher Education Institutes, Anhui Medical University, Hefei City, Anhui Province China; 3 Department of Rehabilitation Medicine, The Second Affiliated Hospital of Anhui Medical University, Hefei City, Anhui Province China; 4 Department of General Surgery, The Second Affiliated Hospital of Anhui Medical University, Hefei City, Anhui Province China; 5 Research Center for Translational Medicine, The Second Hospital of Anhui Medical University, Hefei City, Anhui Province China; 6 Department of Anesthesiology and Pain Medicine, University of California Davis Health, Sacramento, CA USA; 7 Department of Anaesthesiology, LKS Faculty of Medicine, The University of Hong Kong, Pokfulam, Hong Kong SAR China

**Keywords:** Anisodamine, ST36 acupoint, Postoperative nausea and vomiting, Multimodal antiemetic prophylaxis, Laparoscopic sleeve gastrectomy

## Abstract

**Background:**

The use of multimodal pharmacological prophylactic regimes has decreased postoperative nausea and vomiting (PONV) in general but it still occurs in over 60% of female patients after bariatric surgery. This study aimed to evaluate the efficacy of ST36 acupoint injection with anisodamine in prevention of PONV among female patients after bariatric surgery.

**Methods:**

Ninety patients undergoing laparoscopic sleeve gastrectomy were randomly allocated to anisodamine or control group at the ratio of 2:1. Anisodamine or normal saline was injected into Zusanli (ST36) bilaterally after induction of general anesthesia. The incidence and severity of PONV were assessed during the first 3 postoperative days and at 3 months. The quality of early recovery of anesthesia, gastrointestinal function, sleep quality, anxiety, depression, and complications were also evaluated.

**Results:**

Baseline and perioperative characteristics were comparable between two groups. In the anisodamine group, 25 patients (42.4%) experienced vomiting within postoperative 24 h compared with 21 (72.4%) in the control group (relative risk 0.59; 95% confidence interval 0.40–0.85). Time to first rescue antiemetic was 6.5 h in anisodamine group, and 1.7 h in the control group (*P* = 0.011). Less rescue antiemetic was required during the first 24 h in the anisodamine group (*P* = 0.024). There were no differences in either postoperative nausea or other recovery characteristics.

**Conclusions:**

The addition of ST36 acupoint injection with anisodamine significantly reduced postoperative vomiting without affecting nausea in female patients with obesity undergoing laparoscopic sleeve gastrectomy.

**Supplementary Information:**

The online version contains supplementary material available at 10.1007/s00464-023-10037-6.

More than 30% of postoperative patients in general still suffer from postoperative nausea and vomiting (PONV) within 24 h after surgery, and can be as high as 60–80% of high-risk patients based on Apfel score [1, 2]. PONV can cause serious complications such as, aspiration pneumonia, dehydration and wound dehiscence, which further affect postoperative recovery, prolong the hospital length of stay (LOS) and increase health care costs [3]. For patients with obesity, the high incidence of PONV is closely associated with readmissions after bariatric surgery [4], and therefore, preventing PONV is of equal if not more importance to preventing postoperative pain [5].

Previous in vivo and in vitro studies have found that anisodamine can have therapeutic benefits in cardiac arrhythmias, bacteremic shock, arthritis and other conditions [5]. Moreover, as a non-specific cholinergic antagonist, anisodamine inhibits gastrointestinal motility and has protective effects on gastric ulcers and gastrointestinal colic among others [6]. However, there is little work exploring the preventive efficacy of anisodamine in PONV.

There are substantial evidence supporting acupuncture as an adjunctive treatment for postoperative or chemotherapy-induced nausea and vomiting [7, 8]. Repeated acupuncture or continuous electrical acupoint stimulation on Zusanli (ST36) has been used to alleviate PONV [9] and enhance bowel function recovery [10]. However, this repetitive and continuous maneuver is accompanied by the risk of needle dislodgement and requires continual attention throughout surgery. Therefore, reducing the duration and complexity while ensuring efficacy is a priority when using acupuncture to prevent nausea and vomiting.

We hypothesized that adding anisodamine to ST36 acupoint injection would obviate the need for repeated needling and improve its efficacy in preventing PONV. To compare this form of acupuncture to combination prophylaxis, we designed this double-blinded, randomized controlled trial among female patients undergoing laparoscopic sleeve gastrectomy (LSG).

## Materials and methods

### Trial design and ethics

This single-center prospective, randomised, double-blinded study was approved by the Ethics Committee of the Second Affiliated Hospital of Anhui Medical University, Anhui province, China (Chairperson Prof. Yanghua Tian) on December 2, 2021 (Ref YX2021-114) in accordance to the principles of the Declaration of Helsinki. The trial was registered in clinicaltrials.gov (NCT05240482) and carried out at the Second Affiliated Hospital of Anhui Medical University from 20 February to 6 July 2022. Written informed consent was obtained from all participants. The time period of this trial consisted of a 72-h follow-up after bariatric surgery in the hospital and a follow-up period of an additional 3 months after discharge.

The LSG was performed by the same bariatric surgical team, basically referring to the International Sleeve Gastrectomy Expert Panel Consensus Statement [11]. Gastric resection began 4–6 cm proximal to the pylorus and reserved 1 cm lateral to the angle of His, with a calibration 36 F bougie.

### Participants

All adult female patients with an American Society of Anesthesia (ASA) physical status I–III, scheduled for elective LSG surgery were screened. The exclusion criteria: contraindications to acupoint injection, such as a local rash at the site of skin injection or systemic infection, any history of allergic diathesis for drugs used in the study, gastroesophageal reflux, severe obstructive sleep apnea hypopnea syndrome (OSAHS), uncontrolled systemic diseases of the heart, lung, kidney, or liver, coagulation derangements, psychological disorder, current use of medications before surgery that would interference with relevant assessments (including the use of opioids, antiemetics or glucocorticoids), obesity due to a diagnosed endocrine disorder and without consent.

### Randomization and blinding

All participants were randomly assigned to anisodamine group (bilateral ST36 acupoint injection with anisodamine) or control group (bilateral ST36 injection with normal saline) in a 2:1 ratio. The randomization was computer-generated by using www.random.org.

The allocation sequence was concealed until the end of data collection, and the drug or placebo was prepared by the independent researcher thus maintaining blinding of the patients, acupuncturist, outcome assessors.

### Standardized anesthesia management

Dexmedetomidine (0.5 μg kg^−1^) was intravenously infused to provide sedation for the bilateral transverse abdominal plane block (0.33% ropivacaine and dexmedetomidine 1 μg kg^−1^). General anesthesia (GA) was then induced with midazolam (0.05 mg kg^−1^), sufentanil (0.5 μg kg^−1^), propofol (1.5 mg kg^−1^), and rocuronium (1 mg kg^−1^). Following endotracheal intubation, GA was maintained with a continuous infusion of remifentanil (5–15 μg kg^−1^ h^−1^), cisatracurium (0.1–0.2 mg kg^−1^ h^−1^), dexmedetomidine (0.4 μg kg^−1^ h^−1^), and sevoflurane guided by bispectral index. Parecoxib sodium (40 mg i.v.) was administered during disinfection for preemptive analgesia. Patients were administered to the anesthesia intensive care unit (AICU) after extubation. Patient-controlled intravenous analgesia device containing sufentanil (2 μg kg^−1^) and dexmedetomidine (2 μg kg^−1^) was used to alleviate postoperative pain for 48 h. All intravenous anesthetics were calculated by the ideal body weight for each patient.

### ST36 acupoint injection and PONV management

Acupuncture treatment was performed by a licensed acupuncturist who had at least 5 years of acupuncture experience. All participants received bilateral ST36 acupuncture injection with anisodamine (5 mg/1 ml per site) or normal saline (1 ml per site) [12].

All patients received prophylaxis for PONV with dexamethasone (10 mg i.v.) during GA induction, and dorasetron (12.5 mg) at 20 min before the end of surgery [13]. Droperidol (1.25 mg i.v.) followed by dorasetron (12.5 mg i.v.) was administered as rescue medication if patients experienced PONV during AICU stay. All patients were routinely received ondansetron to prevent PONV on postoperative day (POD) 1.

### Outcomes

The primary outcome is the incidence of vomiting within the first 24 h postoperatively.

The secondary outcomes include: the time to the first rescue antiemetic; the incidence of PONV assessed at 2, 6, 48, and 72 h after surgery; the severity of PONV measured by the Verbal Rating Scale (VRS) (none, mild, moderate, or severe) at the same time points; the incidence of postoperative complications; the early recovery outcomes indicators (the time of first drink, ambulate and flatus) and the postoperative LOS. Time to first rescue antiemetic was defined as the time from extubation to the first rescue antiemetic. The changes in gastrointestinal function, sleep-related quality of life, anxiety and depression status from baseline to postoperative 3 months were assessed by the Gastrointestinal Symptom Rating Scale (GSRS) [14], the Pittsburgh Sleep Quality Index Scale (PSQI) [15], Hamilton Anxiety Rating Scale (HAMA) [16] and Hamilton Depression Rating Scale (HAMD) [17], respectively. The higher scores indicate the worse symptoms.

### Statistical analysis

Based on the incidence of postoperative vomiting in our pilot study, the proportions of patients who experienced vomiting within 24 h after surgery in the control group and anisodamine group were 80% and 40% respectively (Table S1). The sample size was calculated to be 84 patients (28 in control and 56 in anisodamine), with a significance level of 5% in the two-sided test and a detection power of 95% which allowing for 15% loss to follow-up.

Continuous variables were presented as mean ± standard deviation (SD) if normally distributed or median (interquartile ranges, IQR) if not. The assumption of data normality was confirmed using a Shapiro-Wilk test. The comparisons between groups were conducted with *t*-tests or Wilcoxon rank sum tests as appropriate. Categorical data were presented as frequencies (percentages) and analyzed with *Chi*-square tests or *Fisher*’s exact tests. Relative risks (RR) with 95% CIs (confidence intervals) were estimated with the log-binomial model for the incidence of postoperative vomiting. For the time to first rescue antiemetic, we calculated the estimates of the median with 95% CIs using the Kaplan-Meier method and the difference between curves were assessed with the log-rank test. Hazard ratio with 95% CI of incidence of vomiting in 24 h after surgery between two groups was estimated with a Cox proportional hazard model. We further assessed their BMI, gastrointestinal function, sleep-related quality of life, anxiety and depression status from baseline to postoperative 3 months with paired *t*-tests or Wilcoxon signed-rank tests. Analyses were performed with SAS version 9.4 (SAS Institute Inc.). Two-sided *P* values less than 0.05 were considered statistically significant.

## Results

**Fig. 1:**
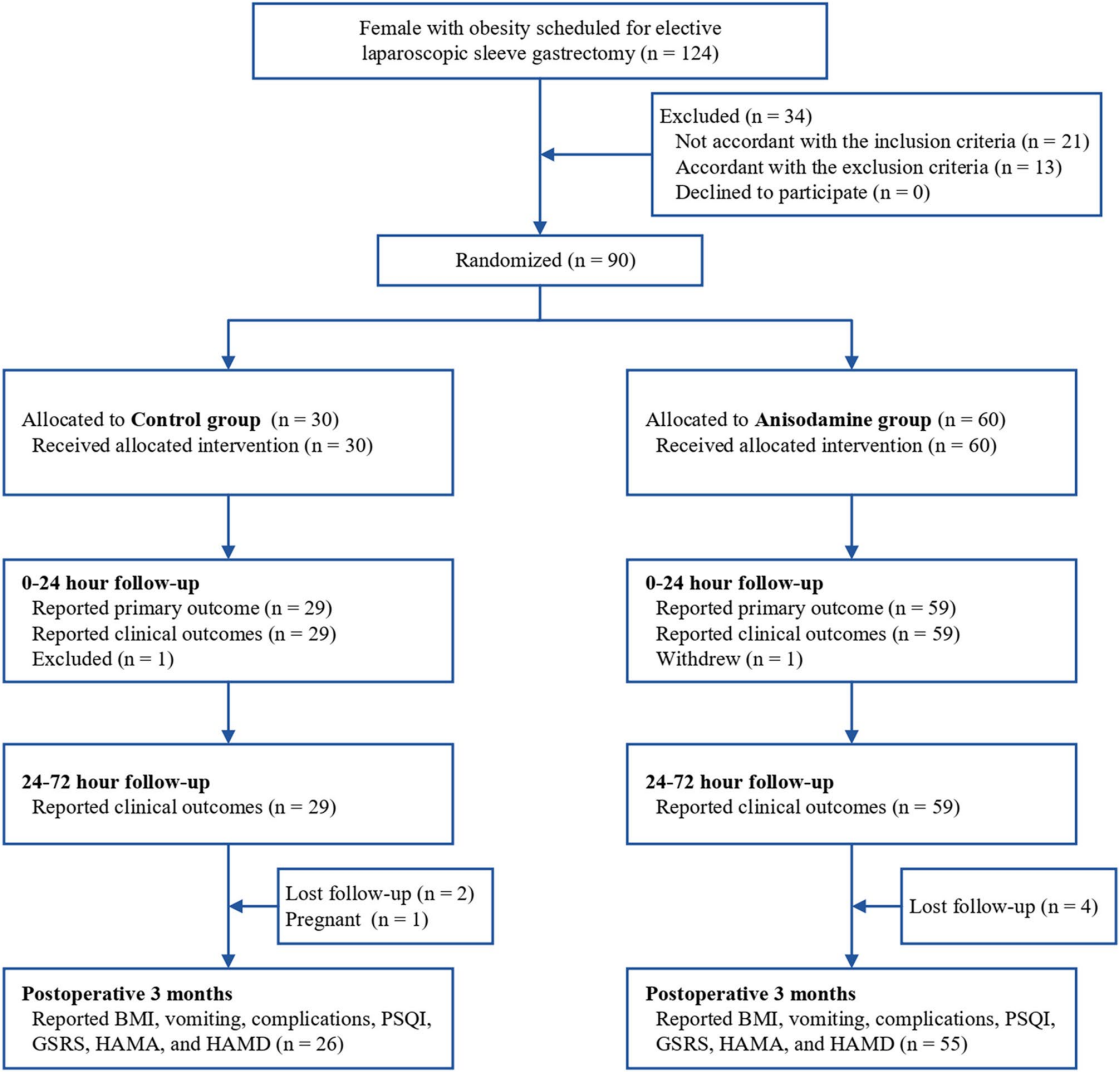
CONSORT flow diagram. *PSQI* Pittsburgh Sleep Quality Index, *GSRS* Gastrointestinal Symptom Rating Scale, *HAMA* Hamilton Anxiety Rating Scale, *HAMD* Hamilton Depression Rating Scale

A total of 124 patients with obesity who underwent LSG were screened and 90 patients were randomly assigned to receive bilateral ST36 acupoint injection with either anisodamine (60 patients) or control (30 patients) (Fig. 1). Eighty-eight patients (59 in the anisodamine group and 29 in the control group) were included in the final analyses as two patients did not complete the postoperative assessments during the first 72 h. Overall baseline and perioperative characteristics of patients were similar between the two groups (Table 1). Moreover, seven patients lost follow-up at postoperative 3 months, and their data were excluded from the followed-up analysis.

**Table 1:** Baseline characteristics and perioperative data for randomised population

	Control group	Anisodamine group	*P*
	(n = 29)	(n = 59)	
Age, year	30.0 (26.0, 32.0)	30.0 (27.0, 34.0)	0.713
BMI, kg/m^2^	36.2 (33.9, 42.5)	37.3 (34.9, 41.7)	0.808
ASA, n (%)			0.912
II, Mild systemic disease	20 (69.0)	40 (67.8)	
III, Severe systemic disease	9 (31.0)	19 (32.2)	
PONV risk score, n (%)			0.148
2	0 (0.0)	4 (6.7)	
3	15 (51.7)	33 (55.9)	
4	14 (48.3)	22 (37.3)	
Hypertension			1.000
No	29 (100.0)	57 (96.6)	
Yes	0 (0.0)	2 (3.4)	
Hyperlipidemia			0.969
No	23 (79.3)	47 (79.7)	
Yes	6 (20.7)	12 (20.3)	
Hyperglycemia			0.052
No	26 (89.7)	42 (71.2)	
Yes	3 (10.3)	17 (28.8)	
Reproductive history			0.829
No	12 (41.4)	23 (39.0)	
Yes	17 (58.6)	36 (61.0)	
Family history of obesity			0.873
No	24 (82.8)	48 (81.4)	
Yes	5 (17.2)	11 (18.6)	
Surgery history, n (%)			0.199
No	16 (55.2)	24 (40.7)	
Yes	13 (44.8)	35 (59.3)	
Propofol, mg	322.2 (276.2, 354.2)	335.7 (292.4, 387.6)	0.284
Remifentanil, µg	1333.0 (1087.5, 1564.0)	1435.5 (1215.0, 1663.5)	0.248
Cisatracurium, mg	6.8 (6.1, 7.9)	7.2 (6.2, 9.3)	0.147
Dexmedetomidine, µg	37.1 (30.3, 44.9)	35.7 (30.6, 44.1)	0.860
Time of anesthesia, min	102.8 ± 17.2	109.2 ± 18.6	0.122
Time of operation, min	84.8 ± 16.2	88.2 ± 15.6	0.339

Data are given as mean ± SD, median (interquartile range, IQR) or percentage (%). *P* values were calculated with *t*-tests, Wilcoxon ran sum tests, *Chi*-square tests, or *Fisher*’s exact tests for continuous and categorical variations as appropriate

*ASA* American Society of Anesthesiology, *PONV* postoperative nausea and vomiting

**Fig. 2:**
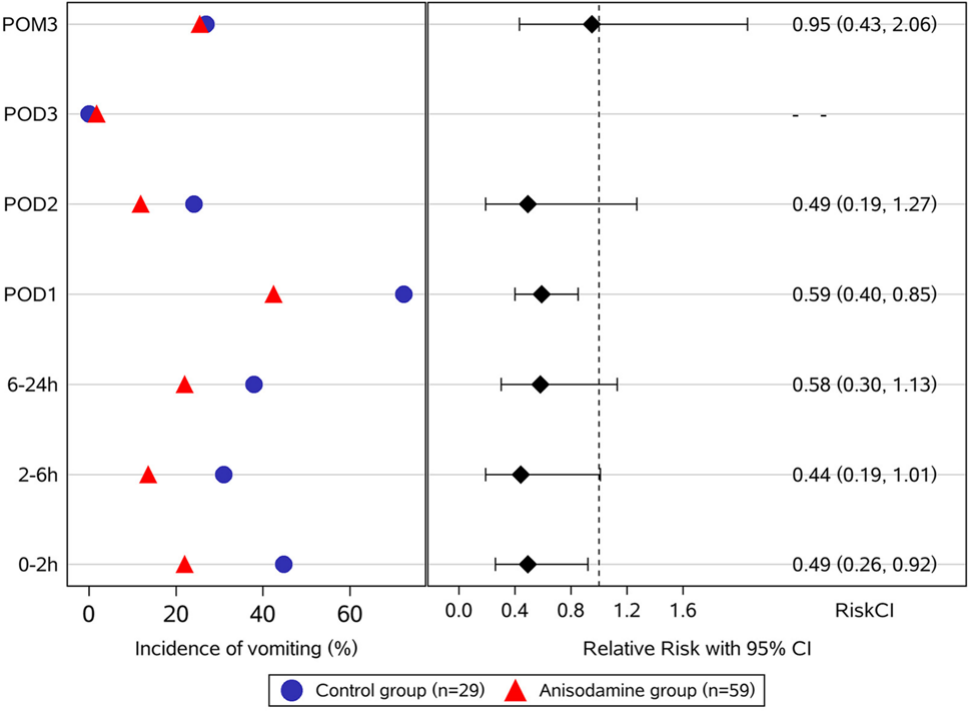
Incidence of postoperative vomiting. *POD* postoperative day, *POM* postoperative month. Relative risks (RR) with 95% CIs (confidence intervals) were calculated using the log-binomial model

The number of patients who experienced vomiting within postoperative 24 h was 25 (42.4%) in the anisodamine group compared with 21 (72.4%) in the control group (relative risk, RR 0.59; 95% CI 0.40–0.85; *P* = 0.005). There was a significant difference in the proportion of vomiting in the first 2 h (22.0% for anisodamine group vs. 44.8% for control group) with RR of 0.49 (95% CI 0.26–0.92; *P* = 0.027). The difference was nearly reached statistical significance until postoperative 6 h (RR 0.44; 95% CI 0.19 to 1.01;* P* = 0.054). There was no difference in the incidence of vomiting between the two groups from 6 h to 3 days postoperatively (Fig. 2 and Table S2).

**Fig. 3:**
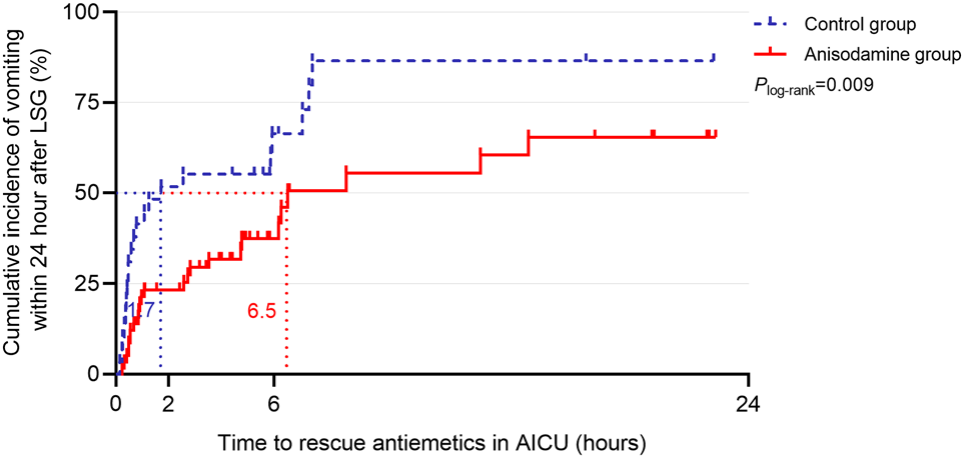
Kaplan Meier plot for time to rescue antiemetic. *AICU* anesthesia intensive care unit, *LSG* laparoscopic sleeve gastrectomy

The time to first rescue antiemetic was significantly longer in the anisodamine group than in the control group (hazard ratio, HR 0.47; 95% CI 0.25–0.89; *P* = 0.011). Median time to first rescue antiemetic was 6.5 h (95% CI 4.5–7.9) in the anisodamine group, and 1.7 h (95% CI 0.0–4.4) in the control group (Fig. 3). There was also less use of rescue dorasetron in the first postoperative 24 h in the anisodamine group than the control group (*P* = 0.024) (Table 2).

**Table 2:** Secondary outcomes of laparoscopic sleeve gastrectomy patients

	Control group	Anisodamine group	*P*
	(n = 29)	(n = 59)	
Incidence of nausea, n (%)			
0–2 h	21 (72.4)	42 (71.2)	0.905
2–6 h	21 (72.4)	32 (54.2)	0.102
6–24 h	21 (72.4)	38 (64.4)	0.453
POD1	26 (89.7)	47 (79.7)	0.225
POD2	12 (41.4)	24 (40.7)	0.950
POD3	1 (3.5)	7 (11.9)	0.164
Severity of PONV, median (IQR)			
0–2 h	3 (0, 3)	1 (0, 3)	0.081
2–6 h	1 (0, 3)	1 (0, 2)	0.074
6–24 h	3 (0, 3)	1 (0, 3)	0.165
POD2	0 (0, 3)	0 (0, 3)	0.984
POD3	0 (0, 0)	0 (0, 0)	0.194
Time in AICU, h	15.3 (5.6, 20.1)	6.6 (4.4, 21.6)	0.912
Droperidol, mg	1.5 (0, 2.0)	1.0 (0, 2.0)	0.318
Dolasetron, mg	0 (0, 12.5)	0 (0, 0)	0.024
First ambulate, h	9.0 (7.0, 19.0)	8.5 (7.0, 19.0)	0.756
First flatus, h	26.0 (23.0, 31.0)	27.0 (23.0, 33.5)	0.880
First drink, h	26.0 (23.0, 41.0)	24.0 (23.0, 26.0)	0.248
Postoperative LOS, d	3.0 (4.0, 5.0)	3.0 (4.0, 6.0)	0.993
QoR-15	114.0 (110.0, 118)	112.0 (106.0, 117.0)	0.130

Data are given as median (interquartile range, IQR) or percentage (%). *P* values were calculated with Wilcoxon ran sum tests, *Chi*-square tests, or *Fisher*’s exact tests for continuous and categorical variations as appropriate

*PONV* postoperative nausea and vomiting, *POD* postoperative day, *AICU* anesthesia intensive care unit, *LOS* length of stay, *QoR-15* Quality of Recovery-15

A similar incidence of postoperative nausea during the first 24 h were seen in the two groups with 47 patients (79.7%) in the anisodamine group and 26 patients (89.7%) in the control group (RR 0.89; 95% CI 0.74–1.06; *P* = 0.195). Also, a similar proportion of nausea were also found in the two groups within postoperative 3 days. The severity of PONV during postoperative 3 days in the anisodamine group and control group showed no significant difference (*P* > 0.05). Moreover, we found no significant difference between the two groups in the early recovery outcomes, the scores of Quality of Recovery-15, as well as the postoperative LOS (Table 2).

Table 3 shows a similar incidence of adverse events in the participants. One patient experienced pulmonary infection on POD2, and two patients experienced of staple-line leak on POD8 and POD12, respectively. The overall rate of reported adverse events in 3 months postoperatively was similar between the anisodamine group (49.0%) and the control group (46.2%). The most common event was alopecia with a total incidence of 43.2% (Table 3).

**Table 3:** Adverse events

	Control group	Anisodamine group
Perioperative period		
Pulmonary infection	1/29 (3.4)	0/59 (0.0)
Staple-line leak	0/29 (0.0)	2/59 (3.4)
Postoperative 3 months		
Alopecia	10/26 (38.5)	25/55 (45.5)
Anemia	1/26 (3.9)	2/55 (3.6)
Staple-line leak	0/26 (0.0)	1/55 (1.8)^a^
Chylous leakage	1/26 (3.9)	0/55 (0.0)
Gastroesophageal reflux	2/26 (7.7)	2/55 (3.6)

Data are given as frequency/number (%)

^a^This patient has suffered from staple-line leak for almost 2 months

Patients reported significant weight loss after 3 months in both groups, with a similar level in %Excess BMI Lost (median 66.8, IQR, 50.5, 84.8 in the anisodamine group and median 66.8, IQR, 45.1, 77.9 in the control group, *P* = 0.459). The total scores of the PSQI, GSRS, and HAMD in both groups also decreased at the end of 3 months postoperatively (POM3) (*P* < 0.05) with a similar degree. However, the scores of HAMA were similar in both groups at baseline and POM3 (Table S3).

## Discussion

In this trial, anisodamine was creatively used on ST36 acupoint injection in female patients during LSG. During the first postoperative 24 h, we successfully observed a significant reduction of the incidence of vomiting, an increase of the time to first rescue antiemetic, as well as less rescue antiemetic in patients with anisodamine injection. It’s noted that ST36 acupoint injection without or with anisodamine had no effect on the early recovery in the hospital and quality of life after discharge.

Since female gender is one of the strongest patients-specific predictors among recognized independent risk factors for PONV [18], we targeted female patients with obesity in this trial. The International Society for the Perioperative Care of the Patient with Obesity (ISPCOP) and the American Society for Metabolic and Bariatric Surgery (ASMBS) recommended using multimodal pharmacologic antiemetic regimens for the prophylaxis of PONV in bariatric surgery, regardless of the preoperative risk scores for PONV [18]. To manage PONV, we used a combination of antiemetics for prophylaxis and rescue. As indicative of the high rate of PONV in bariatric surgery, the proportion of vomiting within postoperative 24 h in the control group was 72.4%, which is similar to the rates reported in the previous study [19]. Compared with female patients undergoing laparoscopic gynecological surgeries, female patients who underwent LSG had a significantly higher occurrence of PONV (23.0% vs. 66.2%) [20]. Operation- and anesthesia-related risk factors may also increase the occurrence of PONV, including intravascular volume deficits, intraoperative opioid consumption, and volatile anesthetic use [13, 21, 22]. The operations were performed with 15 mmHg intra-abdominal pressure insufflated by carbon dioxide in the reverse Trendelenburg position, gastrointestinal hypoperfusion caused by pneumoperitoneum may impair gastrointestinal function and promote the occurrence of PONV [23].

Anisodamine, scopolamine and atropine are common tropine alkaloids with similar pharmacological effects [6], which are widely used for treating spasms, inflammation, septic shock and gastric disorders [24]. Among these, scopolamine, when used as an antiemetics, can provide a similar effect as antihistamines in preventing PONV [25]. However, several randomized controlled trials (RCTs) have shown that the preventive effect of scopolamine against PONV was not significant [26, 27], but can cause dry mouth and blurry vision [28]. Compared to scopolamine, anisodamine has less adverse effect on central nervous system [6] and has similar efficacy as an anti-spasmodic. A multicenter trial indicated that anisodamine and scopolamine have similar efficacy in reliving gastric or intestinal spasm-like pain by inhibiting smooth muscle contractility [29]. The suggested mechanisms are direct acting on the cholinoceptor in the lateral reticular formation of the medulla oblongata, antagonising cholinergic transmission to the vomiting center [30] and producing peripheral and central antimuscarinic actions [31]. Since the cholinoceptors are also located in cardiac vascular and airway smooth muscle, one should carefully monitor any potential side effects with anisodamine [32]. In this trial, no adverse events such as tachyarrhythmia, palpitations, blurred vision, and bowel obstruction, were observed in the anisodamine group. This indicates a better safety margin of anisodamine. In addition, the pilot trial showed that intravenous injection with anisodamine has similar preventive effect as the standard prophylaxis.

Acupuncture, as a nonpharmacologic prophylaxis therapy, has been proposed as an alternative option for multiple multimodal antiemetic therapy because of its safety and affordability [33]. Studies have shown that multiple or prolonged stimulation at one acupoint or multiple acupoints would lower the incidence of PONV. Preoperative bilateral stimulation on PC6 and ST36 acupoints on the day of surgery and the next morning for 30 min lowered the incidence of vomiting by 7.4% in a study of 1655 patients from 12 hospitals undergoing laparoscopic non-gastrointestinal surgery [34]. In female patients undergoing LSG, thrice transcutaneous electrical acupoint stimulation at ST36 acupoint for 30 min (before induction, postoperative 2 h and 6 h) decreased PONV in the first 48 h from 77.4 to 41.9% [9]. In contrast to these complex interventions, we succeeded in reducing the incidence of postoperative vomiting by 30% with a single injection of anisodamine at one acupoint thus simplifying the process while maintaining its effectiveness. Another RCT also found that a combination of ondansetron and ST36 acupoint injections with vitamin B1 provided a significant preventive effect of PONV in laparoscopic surgery [35].

In addition to prophylactic antiemetics, minimizing opioid use through multimodal analgesic regimens can also be effective in reducing the risk of PONV. We established a multimodal analgesic regimen, including perioperative administration of dexmedetomidine and transverse abdominal plane block, in the standardized GA protocol to partially reduce the risk of PONV. A meta-analysis including 697 participants underwent bariatric surgery also indicated that dexmedetomidine significantly reduced PONV in post anesthesia care unit and POD1 with OR from 0.24 to 0.28 [36]. The addition with transversus abdominis plane block was also related with less proportion of PONV [37]. For safety outcomes, a total of three patients (3.4%) were diagnosed with leaks within the two groups, which is higher than the previous reported of less than 1.0% [38, 39]. One of the patients in the anisodamine group has suffered from staple-line leak for almost 2 months. It may be related to her diabetes and might cause in a decreased ability of the tissue to heal. It is reported that, besides complications (i.e., diabetes, sleep apnea), age, surgical technique and operation (i.e. stapling technique, Bougies size) may also cause the high incidence [40, 41], as well as preoperative factors, like weight loss [42]. Therefore, follow-up studies are needed to address this issue further.

## Strengths and limitations

This trial, based on previous studies, simplifies the procedure and duration of acupuncture and provides a comparable preventive effect on postoperative vomiting, which contributes to the popularization of acupuncture in preventing PONV. The results also provide strong evidence using anisodamine for PONV prevention.

This trial has several limitations. First, this study was conducted in a population with a high risk of PONV and achieved a 30% reduction. It was considered unlikely that this intervention could achieve a comparable effect among female patients undergoing other surgical groups. Vomiting in postoperative 24 h still occurred in 42% of patients who received ST36 acupoint injection with anisodamine. Thirdly, compared to standard prophylaxis, ST36 acupoint injection with anisodamine did not reduce the incidence of postoperative nausea. In addition, the leak rate in this study was higher than the previous reported. Further exploration of effective preventions for this population is still needed.

## Conclusions

ST36 acupoint injection with anisodamine had incremental benefit when used as part of combination prophylaxis for reducing PONV. It could be a new option contained in multimodal prophylactic antiemetic regime in female patients undergoing laparoscopic sleeve gastrectomy.

## Supplementary Information

Below is the link to the electronic supplementary material.

Supplementary file1 (DOCX 20 kb)
