# Supplementary material for: ST36 acupoint injection with anisodamine for postoperative nausea and vomiting in female patients after bariatric surgery: a prospective, randomized controlled trial
Source: Surg Endosc. 2023 Apr 24;37(8):5999–6007. doi: 10.1007/s00464-023-10037-6 (PMC10338617; doi:10.1007/s00464-023-10037-6)
Supplement: Supplementary file 1 — Supplementary file1 (DOCX 20 kb) [file 464_2023_10037_MOESM1_ESM.docx]

**Supplementary Information for A Randomised Controlled Trial Evaluating ST36 Acupoint Injection with Anisodamine for Preventing Postoperative Nausea and Vomiting in female patients after Bariatric Surgery**

**Clinical site**

Second Affiliated Hospital of Anhui Medical University

**Data Management and Statistical Center:**

Department of Anesthesiology, Second Affiliated Hospital of Anhui Medical University, Anhui Province, China

**Trial registration**

The protocol has been registered in ClinicalTrials.gov, with approval number NCT05240482.

**Protocol version**

Version number: 1.1. Version data: December 2, 2021.

**Ethics approval**

Ref# YX2021-114.

**Information of pilot study**

In the preliminary study, 15 female patients with obesity were randomly allocated into three groups as following, control group (ST36 injection with normal saline), anisodamine^ST36^ group (ST36 injection with anisodamine) and anisodamine^iv^ group (intravenous injection with anisodamine and ST36 injection with normal saline). Bilateral ST36 injections with either anisodamine (10 mg) or normal saline were performed after general anesthesia induction. Anisodamine administration (10 mg) in intravenous was completed before the end of surgery. The incidences of vomiting within 3 days postoperatively were showed in table below.

| Table S1 Incidence of vomiting within 3 days after laparoscopic sleeve gastrectomy for preliminary test | | | |
| --- | --- | --- | --- |
|  | Control group  (n = 5) | anisodamine^ST36^ group (n = 5) | anisodamine^iv^ group (n = 5) |
| 0-2 h | 2 (0.4%) | 0 (0.0%) | 2 (0.4%) |
| 2-6 h | 2 (0.4%) | 0 (0.0%) | 1 (0.2%) |
| 6-24 h | 4 (0.8%) | 2 (0.4%) | 3 (0.6%) |
| POD1 | 4 (0.8%) | 2 (0.4%) | 4 (0.8%) |
| POD2 | 1 (0.2%) | 2 (0.4%) | 1 (0.2%) |
| POD3 | 0 (0.0%) | 0 (0.0%) | 0 (0.0%) |
| POD, postoperative day; iv, intravenous. | | | |

| Table S2 Incidence of vomiting within 3 days after laparoscopic sleeve gastrectomy. | | | |
| --- | --- | --- | --- |
|  | **Control group (n = 29)** | **Anisodamine group (n = 59)** | ***P*** |
| **0-2 h** | **13 (44.8)** | **13 (22.0)** | **0.028** |
| 2-6 h | 9 (31.0) | 8 (13.6) | 0.051 |
| 6-24 h | 11 (37.9) | 13 (22.0) | 0.116 |
| **POD1** | **21 (72.4)** | **25 (42.4)** | **0.008** |
| POD2 | 7 (24.1) | 7 (11.9) | 0.242 |
| POD3 | 0 (0.0) | 1 (1.7) | 1.000 |
| POM3 | 7 (26.9) | 14 (25.5) | 0.888 |
| POD, postoperative day; POM, postoperative month. | | | |
| Data are given as percentage (%). | | | |
| *P* values were calculated with Chi-square tests, or *Fisher*’s exact tests. | | | |

| Table S3 Characteristics of obese patients before and after laparoscopic sleeve gastrectomy. | | | | |
| --- | --- | --- | --- | --- |
|  |  | **Control group** | **Anisodamine group** | ***P*** |
|  |  | **(n = 26)** | **(n = 55)** |  |
| BMI | |  |  |  |
|  | Baseline | 36.2 (33.9, 42.5) | 37.3 (34.9, 41.7) | 0.808 |
|  | POM3 | 29.5 (27.4, 33.1) | 29.7 (27.4, 32.3) | 0.736 |
|  | %EBMIL | 66.8 (45.1, 77.9)^*^ | 66.8 (50.5, 84.8)^*^ | 0.459 |
| % Total body weight loss | | 21.1 (18.0, 23.3) | 22.3 (19.0, 25.4) | 0.230 |
| PSQI scores | |  |  |  |
|  | Baseline | 2.0 (1.0, 3.0) | 3.0 (1.0, 5.0) | 0.407 |
|  | POM3 | 1.0 (0.0, 2.0) | 1.0 (1.0, 2.0) | 0.510 |
|  | Change | -1.0 (-2.0, 0.0)^*^ | -1.0 (-2.0, 0.0)^*^ | 0.547 |
| GSRS scores | |  |  |  |
|  | Baseline | 0.0 (0.0, 2.0) | 0.0 (0.0, 2.0) | 0.927 |
|  | POM3 | 2.0 (1.0, 4.0) | 2.0 (1.0, 3.0) | 0.513 |
|  | Change | 1.0 (0.0, 3.0)^*^ | 1.0 (0.0, 3.0)^*^ | 0.568 |
| HAMA scores | |  |  |  |
|  | Baseline | 2.0 (1.0, 2.0) | 2.0 (0.0, 2.0) | 0.559 |
|  | POM3 | 1.0 (0.0, 2.0) | 1.0 (0.0, 2.0) | 0.602 |
|  | Change | 0.0 (-2.0, 0.0) | 0.0 (-2.0, 1.0) | 0.694 |
| HAMD scores | |  |  |  |
|  | Baseline | 2.0 (1.0, 3.0) | 1.0 (0.0, 2.0) | 0.459 |
|  | POM3 | 0.0 (0.0, 1.0) | 0.0 (0.0, 1.0) | 0.672 |
|  | Change | -1.0 (-2.0, 0.0)^*^ | -1.0 (-1.0, 0.0)^*^ | 0.281 |
| ^*^, The change from baseline to postoperative 3 months was significant in the same group (*P* < 0.05). | | | | |
| POM, postoperative month; PSQI, Pittsburgh Sleep Quality Index; GSRS, Gastrointestinal Symptom Rating Scale; HAMA, Hamilton Anxiety Rating Scale; HAMD, Hamilton Depression Rating Scale. | | | | |
| Data are given as means with median (interquartile range) or percentage (%). | | | | |
| *P* values were calculated with t-tests or Wilcoxon ran sum tests for continuous variations as appropriate. | | | | |
